# Age determines the prognostic role of the cancer stem cell marker aldehyde dehydrogenase-1 in breast cancer

**DOI:** 10.1186/1471-2407-12-42

**Published:** 2012-01-26

**Authors:** J Sven D Mieog, Esther M de Kruijf, Esther Bastiaannet, Peter JK Kuppen, Anita Sajet, Anton JM de Craen, Vincent THBM Smit, Cornelis JH van de Velde, Gerrit-Jan Liefers

**Affiliations:** 1 Department of Surgery, Leiden University Medical Center, Leiden, The Netherlands; 2 Department of Gerontology & Geriatrics, Leiden University Medical Center, Leiden, The Netherlands; 3 Department of Pathology, Leiden University Medical Center, Leiden, The Netherlands

**Keywords:** Breast cancer, Age, ALDH1, Prognostic factor Part of this work has been presented at the American Association for Cancer Research (AACR) Annual Meeting 2011

## Abstract

**Background:**

The purpose of this study was to compare the expression and the prognostic effect of the breast cancer stem cell marker aldehyde dehydrogenase-1 (ALDH1) in young and elderly breast cancer patients.

**Methods:**

The study population (N = 574) consisted of all early breast cancer patients primarily treated with surgery in our center between 1985 and 1994. Median follow-up was 17.9 years (range: 0.1 to 23.5). Tissue microarray slides were immunohistochemically stained for ALDH1 expression and quantified by two independent observers who were blinded to clinical outcome. Assessment of the prognostic effect of ALDH1 expression was stratified according to age and systemic treatment.

**Results:**

Complete lack of expression of ALDH1 was found in 40% of tumors. With increasing age more tumors showed complete absence of ALDH1 expression (*P *< .001). In patients aged > 65 years, ALDH1 status was not associated with any clinical outcome. Conversely, in patients aged < 65 years, ALDH1 positivity was an independent risk factor of worse outcome for relapse free period (hazard ratio = 1.71 (95% CI, 1.09 to 2.68); *P *= .021) and relative survival (relative excess risks of death = 2.36 (95% CI, 1.22 to 3.68); *P *= .016). Ten-year relative survival risk was 57% in ALDH1-positive patients compared to 83% in ALDH1-negative patients.

**Conclusion:**

ALDH1 expression and its prognostic effect are age-dependent. Our results support the hypothesis that breast cancer biology is different in elderly patients compared to their younger counterparts and emphasizes the importance of taking into consideration age-specific interactions in breast cancer research.

## Background

Age at diagnosis of breast cancer is an important independent prognostic factor. Young age is associated with more aggressive tumors with a relatively high risk of distant metastasis and loco-regional recurrence [1], whereas old age is associated with more indolent tumors [2,3]. Although tumor characteristics differ considerably between age groups (including hormone receptor and human epidermal growth factor receptor 2 (HER2) status), these tumor characteristics can only account for part of the divergence in survival witnessed between age groups [3]. Little is known about the impact and significance of various prognostic and predictive factors in elderly as compared to their younger counterparts. As is the case with randomized trials, elderly are underrepresented in translational studies on molecular markers [4,5]. This caveat is especially worrisome since studies show that the relative survival in elderly breast cancer patient is lower, despite more favorable tumor characteristics, which is probably due to the fact that these patients receive less aggressive treatment [6]. Molecular markers could aid to guide therapy in the fit elderly. Moreover, specific age-interactions might underlie pathophysiological processes in the development of primary breast cancer and subsequent local and distant metastases. Therefore, breast cancer researchers should account for age-specific differences [5].

Recent evidence in tumor biology supports the cancer stem cell theory and may also provide a biological reason for the age-associated survival difference [7]. According to this theory, cancer stem cells, defined as a small subset of tumor cells with stem cell-like features including epithelial-to-mesenchymal transition, have the capacity to self-renewal and differentiation, giving rise to heterogeneous tumor cell population. Various putative markers of breast cancer stem cells have been proposed, including aldehyde dehydrogenase-1 (ALDH1) activity, CD44+/CD24-, CD133, and ITGA6 [7-10]. In particular, ALDH1 expression has shown promise as a clinically relevant prognostic marker [9,11,12]. Moreover, various studies have shown that the subset of cancer stem cells is relatively resistant to chemo- and radiotherapy [13,14]. Thereby, the subpopulation of cancer stem cells can provide both an explanation and a therapeutic target for poor-prognostic, treatment-resistant and recurrent breast cancer.

ALDH1 is a detoxifying enzyme responsible for the oxidation of intracellular aldehydes and thereby confers resistance to alkylating agents [12,15]. This detoxifying capacity might underlie the longevity of stem cells by protecting against oxidative stress. Moreover, ALDH1 may have a role in early differentiation of stem cells and stem cell proliferation through its role in oxidizing retinol to retinoic acid, a modulator of cell proliferation [15]. ALDH1 expression is associated with unfavorable tumor characteristics in breast cancer, such as high grade, absence of hormone receptor expression, positive HER2 status and the basal-like molecular subtype [9,16-18].

To study whether the expression of the breast cancer stem cell marker ALDH1 is associated with age and has an influence on clinical outcome, we analyzed the age-distribution of ALDH1 expression and its prognostic role in young and elderly patients using long-term follow-up data of a cohort of breast cancer patients primarily treated with surgery in our institution.

## Methods

### Study cohort

The patient population comprised all non-metastasized breast cancer patients primarily treated with surgery in the Leiden University Medical Center between 1985 and 1994 with tumor material available (N = 574) [19]. Patients with bilateral tumors or a prior history of cancer (other than basal cell carcinoma or cervical carcinoma *in situ*) were excluded. The following data were known: age, tumor grade, histological type, TNM stage, local and systemic therapy, locoregional or distant tumor recurrence, survival, and expression of estrogen receptor (ER), progesterone receptor (PgR) and human epidermal growth factor receptor 2 (HER2). All tumors were graded according to current pathological standards by an experienced breast cancer pathologist (V.S.). Median follow-up was 17.9 years (range: 0.01 to 23.5). Approval was obtained from the Leiden University Medical Center Medical Ethics Committee. All samples were handled in a coded fashion, according to National ethical guidelines ("Code for Proper Secondary Use of Human Tissue", Dutch Federation of Medical Scientific Societies).

### Assessment of ALDH1 expression

**Figure 1:**
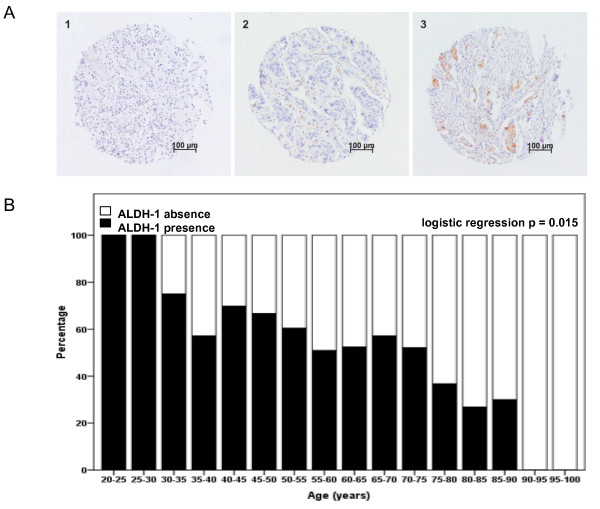
**ALDH1 expression and distribution over age groups**. **A**. Representative photographs of tissue microarray punches of human breast cancer specimens immunohistochemically stained for ALDH1 with representative examples of no staining (left panel), intermediate staining (middle panel) and strong staining (right panel). Bar represents 100 μm. **B**. ALDH1 status according to age (N = 496). Logistic regression *P*-value is shown.

Mouse antibody against ALDH1A1 (611195, BD Biosciences) was used for immunohistochemistry. Tissue sections of 4 μm were cut from a previously constructed tissue microarray of formalin-fixed paraffin-embedded tumors of 574 patients from whom tumor material was available [19]. Immunohistochemical staining was performed according to previously described standard protocols [19]. Human liver tissue slides served as positive control. Negative controls were human liver tissue slides that did undergo the whole immunohistochemical staining without primary antibodies. Microscopic analysis of ALDH1 was assessed independently by two observers in a blinded manner. Absence and presence of ALDH1 activity was classified as 0% and 1-100% staining of tumor cells, respectively (Figure 1A), as was used by others [9,11].

### Statistical analysis

Statistical analyses were performed using the statistical packages SPSS (version 16.0 for Windows, Spps Inc, Chicago, IL, USA) and Stata (version 10.0 for Windows, StataCorp, College Station, TX, USA). Cohen's kappa coefficient was used to assess the inter-observer agreement in quantification of ALDH1 expression. The Cohen's kappa coefficient was 0.81. The χ^2 ^test was used to evaluate associations between various clinicopathological parameters and ALDH1 expression. Relapse-free period was defined as the time from date of surgery until an event (locoregional recurrence and/or a distance recurrence, whichever came first). Relapse-free period is reported as cumulative incidence function, after accounting for death as competing risk [20]. The Kaplan-Meier method was used for survival plotting and log-rank test for comparison of relapse-free period curves. Cox proportional hazard analysis was used for univariate and multivariable analysis for relapse-free period. Relative survival was calculated by the Hakulinen method as the ratio of the survival observed among the cancer patients and the survival that would have been expected based on the corresponding (age, sex, and year) general population. National life tables were used to estimate expected survival. Relative excess risks of death were estimated using a multivariable generalized linear model with a Poisson distribution, based on collapsed relative survival data, using exact survival times.

Analyses were performed for all patients and stratified for age and systemic treatment. Age of 65 years at time of diagnosis was chosen as the cut-off point for age stratification [21]. An interaction term with age and ALDH1 status was introduced in Cox proportional hazard model to assess the interaction in prognostic effects of ALDH1 status for the age groups. Variables with a *P*-value of < .10 in univariate analysis were entered in multivariable analysis.

## Results

### ALDH1 expression in patient cohort

Immunohistochemical data of ALDH1 expression were available for 496 of the 574 patients (86.4%). Of these patients, 326 (65.7%) were < 65 years at diagnosis and 170 (34.3%) were > 65 years at diagnosis. The Cohen's kappa coefficient for inter-observer agreement was 0.81. Complete lack of expression of ALDH1 of any tumor cell was found in 40.4% of the tumors. The association between ALDH1 status and age is shown in Figure 1B. ALDH1 expression was inversely correlated with age (*P *= .0015) and was significantly higher in patients aged < 65 years (65.3%) than in patients aged > 65 years (48.2%; *P *< .001). The association of ALDH1 expression with classic patient, tumor and treatment characteristics is shown in Table 1. In patients aged < 65 years, ALDH1 expression was significantly correlated with high histological grade and positive nodal status. In patients aged > 65 years, ALDH1 expression was significantly correlated with absence of estrogen-receptor expression.

**Table 1:** Association of ALDH1 status with clinicopathological charateristics, stratified by age*.

Characteristic	Patients < 65 years					Patients > 65 years				
	**ALDH1 negative**		**ALDH1 positive**			**ALDH1 negative**		**ALDH1 positive**		

	**N**	**%**	**N**	**%**	***P***	**N**	**%**	**N**	**%**	***P***

**Grade**					0.02					0.79

I	19	17.3	18	8.5		11	12.9	13	16.2	

II	58	52.7	103	48.6		44	51.8	38	47.5	

III	33	30.0	91	42.9		30	35.3	29	36.2	

**Histological type**					0.42					0.20

Ductal	103	92.8	191	90.1		72	84.7	73	91.2	

Lobular	8	7.2	21	9.9		13	15.3	7	8.7	

**Tumor size**					0.34					0.55

T1	45	40.2	74	36.1		27	31.0	24	31.6	

T2	57	50.9	101	49.3		48	55.2	37	48.7	

T3/4	10	8.9	30	14.6		12	13.8	15	19.7	

**Nodal status**					0.02					0.71

Negative	69	62.2	101	47.9		46	56.1	46	59.0	

Positive	42	37.8	110	52.1		36	43.9	32	41.0	

**ER status**					0.61					0.01

Negative	43	41.0	91	44.0		16	19.0	30	38.0	

Positive	62	59.0	116	56.0		68	81.0	49	62.0	

**PgR status**					0.77					0.08

Negative	48	47.1	84	40.6		26	31.3	35	44.9	

Positive	54	52.9	123	59.4		57	68.7	43	55.1	

**HER2 status**					0.99					0.80
Negative	67	87.0	153	86.9		61	93.8	64	92.8	

Positive	10	13.0	23	13.1		4	6.2	5	7.2	

*Missing data not shown.

Abbreviations: *N *number of patients; *ALDH1 *aldehyde dehydrogenase 1; *ER *estrogen receptor; *PgR *progesterone receptor; *HER2 *human epidermal growth factor receptor 2

### Impact of ALDH1 on survival

**Figure 2:**
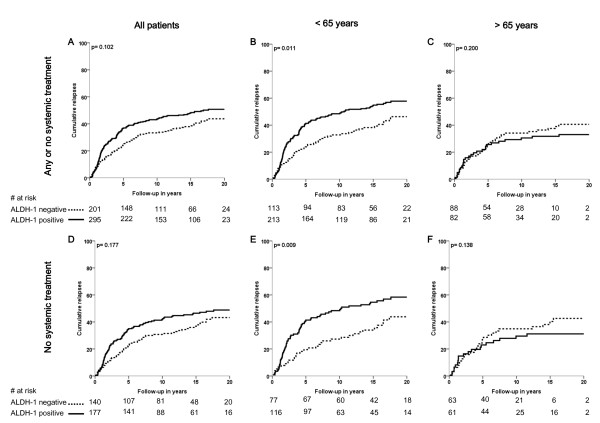
**Relapse free period according to ALDH1 status for all patients (A, D), for patients aged < 65 years (B, E) and for patients aged > 65 years (C, F); and for patients that received any or no systemic therapy (A-C) and for patients that did not receive systemic therapy (D-F)**. Log-rank *P*-values are shown in each graph.

**Figure 3:**
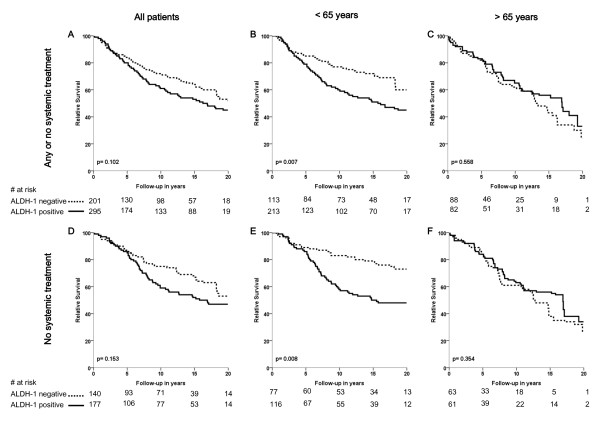
**Relative survival according to ALDH1 status for all patients (A, D), for patients aged < 65 years (B, E) and for patients aged > 65 years (C, F); and for patients that received any or no systemic therapy (A-C) and for patients that did not receive systemic therapy (D-F)**. Log-rank *P*-values are shown in each graph.

The association of ALDH1 status with relapse-free period and relative survival is shown in Figures 2 and 3, respectively. Analysis of relapse-free period showed a trend towards a significant association between ALDH1 status and clinical outcome for the whole population (*P *= .10; Figure 2A, D). In the group of patients aged younger than 65 years, a strong association was found between ALDH1 expression and poor clinical outcome (*P *= .01; Figure 2B). In the subgroup of younger patients who did not receive any systemic treatment, a comparable association was found (*P *= .009; Figure 2E). In this group, 52% of patients with ALDH1-positive tumors was relapse-free at 10 years follow-up compared to 72% of patients with ALDH1-negative tumors (absolute difference = 20%). Conversely, in the elderly patients, no association was found between ALDH1 status and clinical outcome (*P *= .20; Figure 2C, F). Interaction analysis demonstrated a statistically significant difference in the prognostic effect of ALDH1 status in young and elderly patients (*P *= .007).

Analysis of relative survival showed a similar pattern as for relapse-free period: a strong association between ALDH1-positive tumors and poor relative survival in the younger patient group (Figure 3B, E) and no association between ALDH1 status and relative survival for elderly patients (Figure 3C, F). In the subgroup of younger patients who did not receive any systemic treatment, the 10-year relative survival rate was 57% in patients with ALDH1-positive tumors compared to 83% in patients with ALDH1-negative tumors (absolute difference = 26%, *P *= .008; Figure 3E). Interaction analysis demonstrated a statistically significant difference between the prognostic effect of ALDH1 status in young and elderly patients (*P *= 0.047).

Multivariable analyses were conducted for the patient groups that did not receive systemic treatment (276 young patients and 154 elderly patients). ALDH1 status remained an independent prognostic factor in the young patient group for both relapse-free period (hazard ratio = 1.71; 95% CI, 1.09 to 2.68; *P *= .021; Table 2) and relative survival (relative excess risks of death = 2.36; 95% CI, 1.22 to 3.68; *P *= .016; Table 3).

**Table 2:** Univariate and multivariable analysis for relapses free period stratified by age for patients naive to systemic treatment

Characteristic	Patients < 65 years							Patients > 65 years						
		**Univariate analysis**			**Multivariable analysis**				**Univariate analysis**			**Multivariable analysis**		

	**N**	**HR**	**95% CI**	***P***	**HR**	**95% CI**	***P***	**N**	**HR**	**95% CI**	***P***	**HR**	**95% CI**	***P***

**Age**				.74										

< 40	33	1						0						

40-50	98	1.10	0.62-1.96					0						

50-60	101	1.29	0.73-2.29					0						

> 60	44	1.29	0.66-2.52					154						

**Grade**				.03			0.21				.01			.29

I	31	1			1			24	1			1		

II	131	1.45	0.76-2.75		1.06	0.40-2.78		74	1.73	0.66-4.58		1.30	0.48-3.58	

III	75	2.17	1.12-4.20		1.54	0.58-4.11		44	3.66	1.39-9.61		2.01	0.70-5.75	

**Histological type**				.54							.53			

Ductal	223	1						124	1					

Other	15	1.24	0.63-2.45					18	0.74	0.30-1.87				

**Tumor stage**				< .001			0.05				.03			.90

pT1	137	1			1			59	1			1		

pT2	109	1.66	1.16-2.37		1.60	1.01-2.55		71	2.23	1.22-4.09		1.16	0.56-2.40	

pT3/4	22	2.73	1.57-4.76		2.04	1.07-3.88		18	2.11	0.82-5.45		1.23	0.42-3.63	

**Nodal stage**				< .001			< 0.001				< .001			< .001

Negative	204	1			1			109	1			1		

Positive	69	4.25	3.02-5.99		4.44	2.89-6.82		36	3.94	2.28-6.82		3.33	1.77-6.24	

**ER status**				.59							.22			

Negative	87	1						40	1					

Positive	126	0.90	0.66-1.32					95	1.53	0.78-2.98				

**PgR status**				.78							.78			

Negative	84	1						55	1					

Positive	123	0.95	0.64-1.40					81	1.08	0.61-1.92				

**HER2 status**				.26							.05			

Negative	143	1						105	1					

Positive	19	1.44	0.76-2.71					4	0.05	0.00-23.3				

**ALDH1 status**				.01			0.02				.14			

Negative	77	1			1			63	1					

Positive	116	1.75	1.14-2.68		1.71	1.09-2.68		61	0.64	0.35-1.16				

Abbreviations: *N *number of patients; *HR *hazard ratio; *ER *estrogen receptor; *PgR *progesterone receptor; *HER2 *human epidermal growth factor receptor 2; *ALDH1 *aldehyde dehydrogenase 1

**Table 3:** Univariate and multivariable analysis for relative survival stratified by age for patients naive to systemic treatment

Characteristic	Patients < 65 years							Patients > 65 years						
		**Univariate analysis**			**Multivariable analysis**				**Univariate analysis**			**Multivariable analysis**		
	
	**N**	**RER**	**95% CI**	***P***	**RER**	**95% CI**	***P***	**N**	**RER**	**95% CI**	***P***	**RER**	**95% CI**	***P***

**Age**				.06			.10							

< 40	33	1			1			0						

40-50	98	1.03	0.51-2.08		0.69	0.21-2.24		0						

50-60	101	1.52	0.76-3.03		1.38	0.46-5.18		0						

> 60	44	2.16	0.99-4.66		1.93	0.56-6.57		154						

**Grade**				.03			.95				.34			

I	31	1			1			24	1					

II	131	2.23	0.80-6.28		1.24	0.31-5.03		74	3.10	0.43-22.4				

III	75	3.48	1.22-9.94		1.27	0.30-5.45		44	4.23	0.56-31.8				

**Histological type**				.80							.37			

Ductal	223	1						124	1					

Other	15	1.12	0.46-2.71					18	1.49	0.62-3.61				

**Tumor stage**				< .001			.33				.01			.09

pT1	137	1			1			59	1			1.00		

pT2	109	2.67	1.41-3.64		1.42	0.73-2.76		71	5.47	0.83-35.9		4.26	0.71-25.4	

pT3/4	22	4.26	2.29-7.94		1.79	0.81-3.95		18	13.2	1.90-92.1		7.90	1.18-52.9	

**Nodal stage**				< .001			< .001				.02			.43

Negative	204	1			1			109	1			1.00		

Positive	69	5.03	3.32-7.62		5.82	3.16-10.7		36	2.76	1.20-6.35		1.48	0.56-3.92	

**ER status**				.11							.91			

Negative	87	1						40	1					

Positive	126	0.70	0.45-1.09					95	0.95	0.42-2.17				

**PgR status**				.25							.58			

Negative	84	1						55	1					

Positive	123	0.77	0.49-1.21					81	0.80	0.36-1.77				

**HER2 status**				.19							.89			

Negative	143	1						105	1					

Positive	19	1.57	0.80-3.09					4	0.85	0.09-8.40				

**ALDH1 status**				.008			.02				.35			

Negative	77	1			1			63	1					

Positive	116	2.12	1.22-3.68		2.36	1.17-4.73		61	0.68	0.30-1.53				

Abbreviations: *N *number of patients; *RER *relative excess risk; *ER *estrogen receptor; *PgR *progesterone receptor; *HER2 *human epidermal growth factor receptor 2; *ALDH1 *aldehyde dehydrogenase 1

## Discussion

In this study, we demonstrated that the presence of ALDH1 expression is significantly higher in young breast cancer patients than in elderly patients. Moreover, we demonstrated that ALDH1 expression is an independent risk factor for decreased survival in young breast cancer patients, but not in elderly patients.

To the best of our knowledge, we are the first to show that expression of ALDH1 in breast cancer is age-dependent. A corresponding difference in the number of cancer stem cells might provide an explanation for known differences in clinical outcome between young and elderly breast cancer patients. A potential strength of our study is that it includes consecutive patients from one center, not biased by being part of a clinical trial. The age restriction of the majority of clinical trials prohibits inclusion of patients older than 70 year and, indeed, less than 10% of clinical trial participants is older than 65 years [22]. In our study, 34% of patients were 65 years or older at diagnosis of breast cancer. Therefore, our study was not hampered by lack of statistical power to analyse the effect of ALDH1 in the elderly.

We showed that ALDH1 expression has a qualitative age interaction effect. In our study, ALDH1 is a predictor of poor prognosis in young patients, but ALDH1 did not influence clinical outcome in elderly patients. Recently, Zhou and colleagues pooled the available data on the prognostic role of ALDH1 activity in breast cancer [18]. Their meta-analysis demonstrated that ALDH1 activity as assessed by immunohistochemistry was significantly associated with worse overall survival (unadjusted pooled relative risk, 2.83; 95% CI, 2.16 to 3.67; four patient cohorts including 1,158 patients) [18]. However, the authors did not stratify for age. In other studies, no interaction was found between ALDH1 expression and age [9,14,17]. However, in these studies, an age of 40 or 50 year was used as a cut-off for age stratification. We used 65 years as a cut-off point as this may better match with the bimodal age distribution of breast cancer, which suggests that breast cancer may be characterized by early- and late-onset tumor types with modes near ages 50 and 70 years [5,23]. As argued by Anderson et al., these modal ages do not suggest a sharp division of distinctive tumor categories, but rather reflect central tendencies for the age distributions of biologically distinct cancer populations [5,23]. In line with this bimodal age distribution, a biological explanation of the qualitative age-interaction of the prognostic effect of ALDH1 expression might be that of a changing micro-environment in elderly patients, which may result in hampered signal transduction between tumor stem cells and the micro-environment. Moreover, changes in metabolic processes might limit the role of tumor stem cells in elderly patients. Increasing evidence from the field of epigenetics demonstrates that hypermethylation-induced repression of genes required for stem cell differentiation is linearly associated with age [24]. This suggests that, with increasing age, the role of tumor stem cells becomes more limited. Notwithstanding the need to clarify the underlying mechanism, this new finding on the age-dependent role of ALDH1 activity warrants further validation and underlines the need of age stratification when assessing biomarkers and new therapies for breast cancer patients.

A potential limitation of our study is the choice of antibody that was used for immunohistochemical detection of ALDH1 expression, which is specific for the ALDH1A1 isoform. This antibody has been generally used in studies investigating the role of ALDH1 in breast cancer patients [9,11,12,14]. In a recent study, Marcato et al. investigated the expression of the different ALDH1 isoforms in breast cancer stem cells, breast cancer cell lines and fixed human breast cancer tissue using various techniques [25]. They found that ALDH1A3 expression correlated better with ALDH1 activity and with tumor grade, metastasis and cancer stage [25,26]. Therefore, future research studying breast cancer stem cells should incorporate ALDH1A3 expression in order to determine its role as a potential marker of cancer stem cell activity.

## Conclusion

In conclusion, we demonstrated that expression of the putative breast cancer stem cell marker ALDH1 and its prognostic effect are age-dependent in breast cancer patients. We demonstrate, for the first time, the different prognostic impact of a molecular marker in elderly, which suggests that fundamentally different biological mechanisms underlie age-related breast cancer prognosis. Our results support the hypothesis that breast cancer biology of elderly patients and their younger counterparts is distinct and emphasizes the importance of analyzing and reporting age-specific effects in breast cancer research.

## Abbreviations

ALDH1: Aldehyde dehydrogenase-1; ER: Estrogen receptor; HER2: Human epidermal growth factor receptor 2; PgR: Progesterone receptor.

## Competing interests

None of the authors who contributed to this article have any financial or personal relationships with people or organizations that could inappropriately influence the data published.

## Authors' contributions

SM conceived of the study and designed the study, quantified ALDH1 expression and drafted the manuscript. EK quantified ALDH1 expression, performed statistical analysis and helped to draft the manuscript. EB participated in statistical analysis and helped to draft the manuscript. PK participated in coordination of the study and helped to draft the manuscript. AS carried out the immunohistochemical analysis. AC helped to draft the manuscript. VS supervised the quantification of ALDH1 expression and helped to draft the manuscript. CV participated in coordination of the study and helped to draft the manuscript. GJL participated in the design and coordination of the study and helped to draft the manuscript. All authors read and approved the final manuscript.

## Pre-publication history

The pre-publication history for this paper can be accessed here:

http://www.biomedcentral.com/1471-2407/12/42/prepub
